# Intracellular Trafficking of Guanylate-Binding Proteins Is Regulated by Heterodimerization in a Hierarchical Manner

**DOI:** 10.1371/journal.pone.0014246

**Published:** 2010-12-07

**Authors:** Nathalie Britzen-Laurent, Michael Bauer, Valeria Berton, Nicole Fischer, Adrian Syguda, Simone Reipschläger, Elisabeth Naschberger, Christian Herrmann, Michael Stürzl

**Affiliations:** 1 Division of Molecular and Experimental Surgery, University Medical Center Erlangen, Friedrich-Alexander University of Erlangen-Nuremberg, Erlangen, Germany; 2 Physical Chemistry I, Faculty of Chemistry and Biochemistry, Ruhr University Bochum, Bochum, Germany; Institut Européen de Chimie et Biologie, France

## Abstract

Guanylate-binding proteins (GBPs) belong to the dynamin family of large GTPases and represent the major IFN-γ-induced proteins. Here we systematically investigated the mechanisms regulating the subcellular localization of GBPs. Three GBPs (GBP-1, GBP-2 and GBP-5) carry a C-terminal CaaX-prenylation signal, which is typical for small GTPases of the Ras family, and increases the membrane affinity of proteins. In this study, we demonstrated that GBP-1, GBP-2 and GBP-5 are prenylated *in vivo* and that prenylation is required for the membrane association of GBP-1, GBP-2 and GBP-5. Using co-immunoprecipitation, yeast-two-hybrid analysis and fluorescence complementation assays, we showed for the first time that GBPs are able to homodimerize *in vivo* and that the membrane association of GBPs is regulated by dimerization similarly to dynamin. Interestingly, GBPs could also heterodimerize. This resulted in hierarchical positioning effects on the intracellular localization of the proteins. Specifically, GBP-1 recruited GBP-5 and GBP-2 into its own cellular compartment and GBP-5 repositioned GBP-2. In addition, GBP-1, GBP-2 and GBP-5 were able to redirect non-prenylated GBPs to their compartment in a prenylation-dependent manner. Overall, these findings prove *in vivo* the ability of GBPs to dimerize, indicate that heterodimerization regulates sub-cellular localization of GBPs and underscore putative membrane-associated functions of this family of proteins.

## Introduction

Guanine nucleotide binding proteins (G proteins) are involved in important cellular processes, including signal transduction, translation, vesicle trafficking and exocytosis [1]. G proteins function as GDP/GTP-regulated switches with an “inactive” GDP-bound state and an “active” GTP-bound state [2]. The subcellular trafficking of GTPases coordinates their activity in time and space and regulates their function. The mechanisms controlling the localization of the proteins from the Ras family of small GTPases have been extensively studied. Small GTPases display a general affinity for localization to cellular membranes, which depends on structural signals [3]. More specifically, GTPases are characterized by the presence of a carboxyl-terminal “CaaX” motif for prenylation (where “C” represents cysteine, “a” is an aliphatic amino acid and “X” is any amino acid). Prenylation is a posttranslational modification leading to the attachment of a lipid hydrophobic moiety which allows the protein to anchor to cellular membranes. The process of prenylation involves the addition of a C15 farnesyl or a C20 geranylgeranyl isoprenoid to the cysteine residue of the CaaX motif by a farnesyl transferase (FTase) or geranylgeranyl transferase type I (GGTase-I), respectively [4]. Additional structural signals have been described that further increase membrane affinity; these signals include the presence of a cluster of polybasic amino acid residues directly upstream of the CaaX box (K-Ras 4B) or of one or two palmitoylation sites (H-Ras, N-Ras and K-Ras 4A) [5], [6]. Some other proteins, such as Arf, undergo N-terminal myristoylation [7].

In contrast to small GTPases, relatively little is known about the mechanisms mediating the localization of GTPases from the family of guanylate-binding proteins (GBPs). Human GBPs belong to the dynamin family of large GTPases [8]. Seven GBPs have been identified in humans (GBP-1 to -7) [9] and five of them (GBP-1, GBP-2, GBP-3, GBP-4 and GBP-5) have been shown to be highly induced by IFN-γ in eukaryotic cells [10], [11]. GBP-1 is the best-characterized member of the GBP family and mediates cellular responses to IFN-γ in infection and inflammation, suggesting that GBP-1 may be an important component of the innate immune response [12]–[18].

Structurally, GBPs have a molecular weight of 67–73 kDa, display a high degree of homology and share highly conserved GTP-binding or hydrolysis domains [19], [20]. Among human GBPs, three members (GBP-1, GBP-2 and GBP-5) possess a “CaaX” prenylation motif at their C-terminal end, which suggests an affinity for cellular membranes. Previous biochemical studies revealed that GBP-1 and GBP-5 are able to form dimers and even tetramers *in vitro*, and that they display an oligomerization-dependent activation of GTP hydrolysis [21], [22]. This feature is characteristic of the family of large GTPases [23]. In the case of dynamin, it has been shown that the dimerization upon GTP binding regulates the membrane association of the protein [24], [25].

In the present study we investigated whether prenylation and dimerization regulate the intracellular localization and the membrane association of the IFN-γ-induced GBPs. Considering the high homology of GBPs and the fact that GBP-1 to GBP-5 are co-expressed in the cell in response to IFN-γ, we hypothesized that GBPs might also be able to form heterodimers, which may in turn influence the localization of the proteins.

## Results

### Prenylation and GBP localization

**Figure 1:**
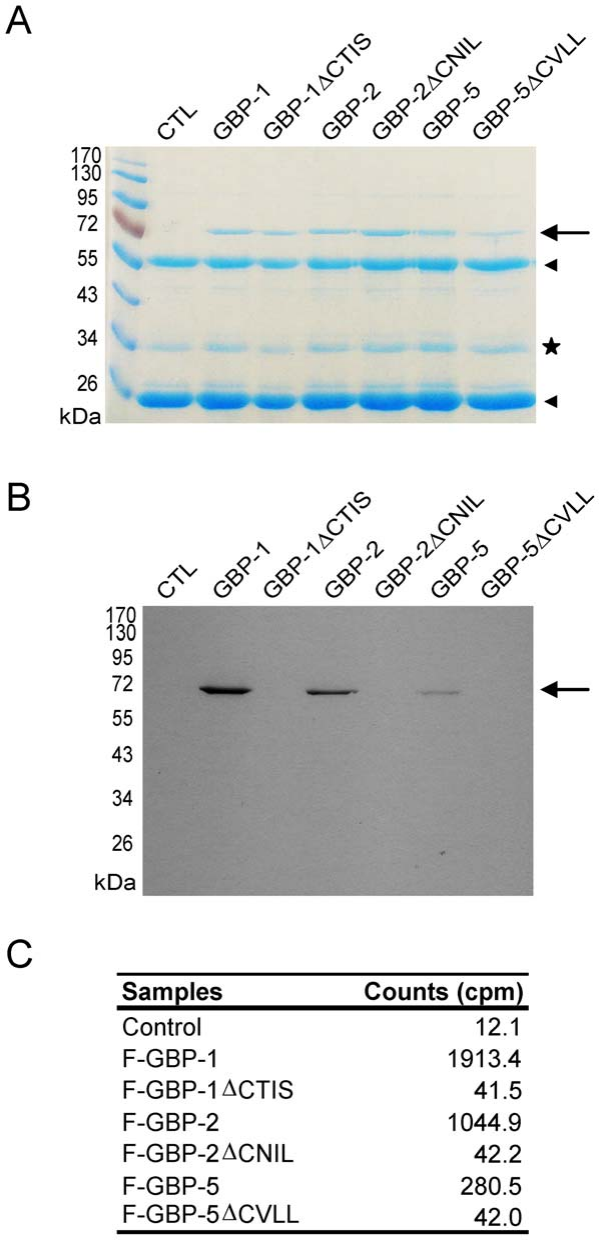
Human GBP-2 and GBP-5 are prenylated *in vivo*. HeLa cells transfected with the empty vector (control) or HeLa expressing Flag-GBP-1, -2 or -5, as well as the respective ΔCaaX mutants, were treated with 200 µCi of [^3^H]-MVA for 20 h before harvesting. Proteins were extracted, immunoprecipitated with an anti-Flag antibody and separated by 10% SDS-PAGE. (**A**) Coomassie stained gel. GBPs, GBP mutants are indicated (arrow), as well as the heavy and light chains of the anti-Flag antibody (arrowheads). An unspecific co-precipitated protein is visible (star) (**B**) Fluorogramm of the gel shown in 1*A*. The gel was exposed for 3 weeks. (**C**) Activity measurements of the immunoprecipitates (in cpm, counts per minutes; one fifth of the precipitates was used for quantitative determination).

Computer-assisted analyses indicated that GBP-1 is modified by the farnesyltransferase (FTase) and suggested that GBP-2 and GBP-5 are modified by geranylgeranyltransferase I (GGTase-I). In addition to its prenylation motif, GBP-1 possesses a region rich in polybasic amino acids (KMRRRK at positions 582 to 587, abbreviation pB) directly upstream of the CaaX box, which might increase the affinity of GBP-1 for cellular membranes [26], [27]. No evidence of myristoylation or C-terminal palmitoylation, which have been shown to regulate the localization of small GTPases, was found in GBPs. GBP-1 has been previously shown to be prenylated *in vivo* [28]. Here we investigated whether GBP-2 and GBP-5 are prenylated *in vivo*. Flag-tagged GBP-2 and GBP-5, and as controls Flag-GBP-1 and the corresponding CaaX mutants (Flag-GBP-1ΔCTIS, Flag-GBP-2ΔCNIL and Flag-GBP-5ΔCVLL), were expressed in HeLa cells. Cells were incubated with [^3^H]-MVA, a precursor of farnesyl and geranylgeranyl pyrophosphates, in presence of Lovastatin, an inhibitor of the endogenous mevalonate metabolic pathway. Flag-tagged proteins were then immunoprecipitated, separated by SDS-PAGE and stained with Coomassie blue solution (Fig. 1A). Flag-GBP-1, Flag-GBP-2 and Flag-GBP-5 incorporated [^3^H]-MVA while the respective CaaX deletion mutants did not, as shown by the fluorography of the gel (Fig. 1*B*) and quantitative determination of the radioactivity by scintillation counting (Fig.1*C*).

**Figure 2:**
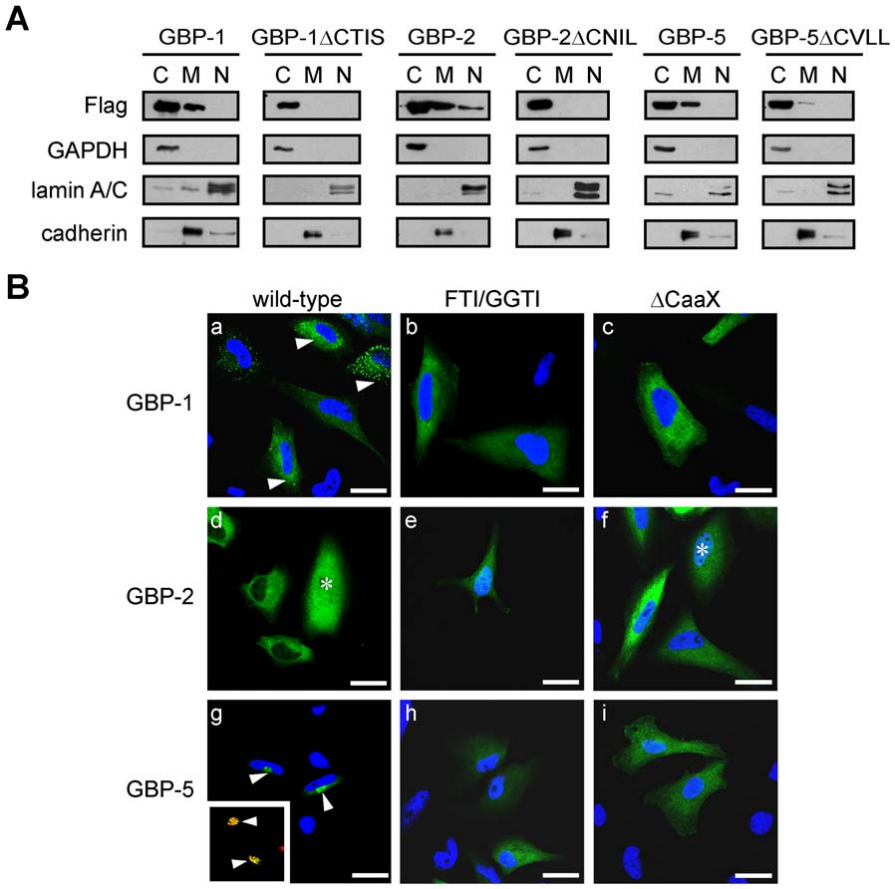
The CaaX box of GBP-1, GBP-2 and GBP-5 is necessary for the association of the proteins with membranes. (**A**) HeLa cells expressing Flag-GBP-1, -2 or -5, as well as the respective ΔCaaX mutants, were fractionated into cytosolic, membranous and nuclear fractions which were analyzed by western blot. GAPDH was used as a marker of the cytosolic fraction, lamin A/C as a marker of the nuclear fraction and cadherin as a marker for the membrane fraction. (**B**) HeLa cells were transiently transfected with Flag-GFP-GBP-1, Flag-GFP-GBP-2 and Flag-GFP-GBP-5. Cells were either left untreated (a, d, g) or treated with FTI (b, inhibition of GBP-1 farnesylation) and GGTI (e and h, inhibition of GBP-2 and GBP-5 geranylgeranylation, respectively). The presence of GBP-2 in the nucleus is indicated by an asterisk (d and f). Alternatively, HeLa cells were transiently transfected with plasmids expressing GFP-GBP fusion proteins lacking the CaaX box: (c) Flag-GFP-GBP-1ΔCTIS, (f) Flag-GFP-GBP-2ΔCNIL and (i) Flag-GFP-GBP-5ΔCVLL. The insert in (e) shows a co-localization (yellow, arrowhead) between GFP-GBP-5 in green and the Golgi marker GM130 in red. Nuclei were counterstained with DAPI and the Golgi apparatus was stained with an anti-GM130 antibody and an anti-mouse-Alexa546 secondary antibody. Scale bars = 25 µm.

The effects of prenylation on the cellular distribution of GBP-1, GBP-2 and GBP-5 were further investigated. Membrane association of Flag-tagged GBP-1, GBP-2, GBP-5 or the corresponding mutants lacking the prenylation motif (ΔCTIS, ΔCNIL and ΔCVLL) was analyzed using cell fractionation and western blot analysis (Fig. 2*A*). All three GBPs showed an association with membranes, whereas their respective CaaX deletion mutants were either not detectable in the membranous fraction (GBP-1ΔCTIS and GBP-2 ΔCNIL) or were present in significantly reduced amounts (GBP-5ΔCVLL). GBP-2 was also detected in the nuclear fraction, confirming a previous localization study [11]. In contrast, GBP-3 and GBP-4 were not associated with membranes (Figure S1 *A*). In agreement with previous localization studies, GBP-3 was exclusively detected in the cytosolic fraction and GBP-4, both in the cytosol and the nuclear fractions [11]. These results showed that presence of the CaaX motif is required for the association of GBPs with membranes.

The localization of GFP-fused GBP-1, GBP-2 and GBP-5 was also investigated at the single cell level using confocal fluorescence microscopy (Fig. 2*B*). The localization of the various GBPs was confirmed after transfection of Flag-tagged GBP-1, -2 or -5 and anti-Flag fluorescence staining, thus excluding an effect of GFP on localization (Fig. S1 *B*). GBP-1 was observed in the cytoplasm of cells in a diffuse or a granular pattern, or both [Fig. 2*B*(a), arrowhead], confirming our previous results [11]. In the presence of FTase inhibitor (FTI) [Fig. 2*B*(b)], but not GGTase-I inhibitor (GGTI) (Fig. S1 *C*), GBP-1 was found to be evenly distributed throughout the cytoplasm. In the case of GBP-1, the absence of prenylation abrogated both membrane association and granular appearance, suggesting that the protein might be associated with the membranes of vesicular structures. Accordingly, the term “vesicle-like” will be used in the remainder of the paper to describe the granular distribution pattern of GBP-1. GBP-2 was distributed evenly throughout the cytoplasm of the cells [Fig. 2*B*(d)] and was also detected in the nucleus [Fig. 2*B*(d), asterisk]. Treatment with either GGTI or FTI did not influence the localization pattern of GBP-2 [Fig. 2*B*(e) and Fig. S1 *C*]. GBP-5 was concentrated in the perinuclear region [Fig. 2*B*(g), arrowheads] and co-localized with the Golgi marker GM130 [Fig. 2*B*(g), insert, arrowheads] [29]. Treatment with GGTI [Fig. 2*B*(h)] but not FTI (Fig. S1 *C*) inhibited the accumulation of GBP-5 at the Golgi, which confirmed that the protein is geranylgeranylated. Expression of mutants of GBP-1, -2 and -5, devoid of the CaaX motif, confirmed the results obtained with the prenylation inhibitors. GBP-1ΔCTIS and GBP-5ΔCVLL localized evenly throughout the cytoplasm, whereas GBP-2ΔCNIL was distributed similarly to the wild-type protein [Fig. 2*B*(c, f, i)].

**Figure 3:**
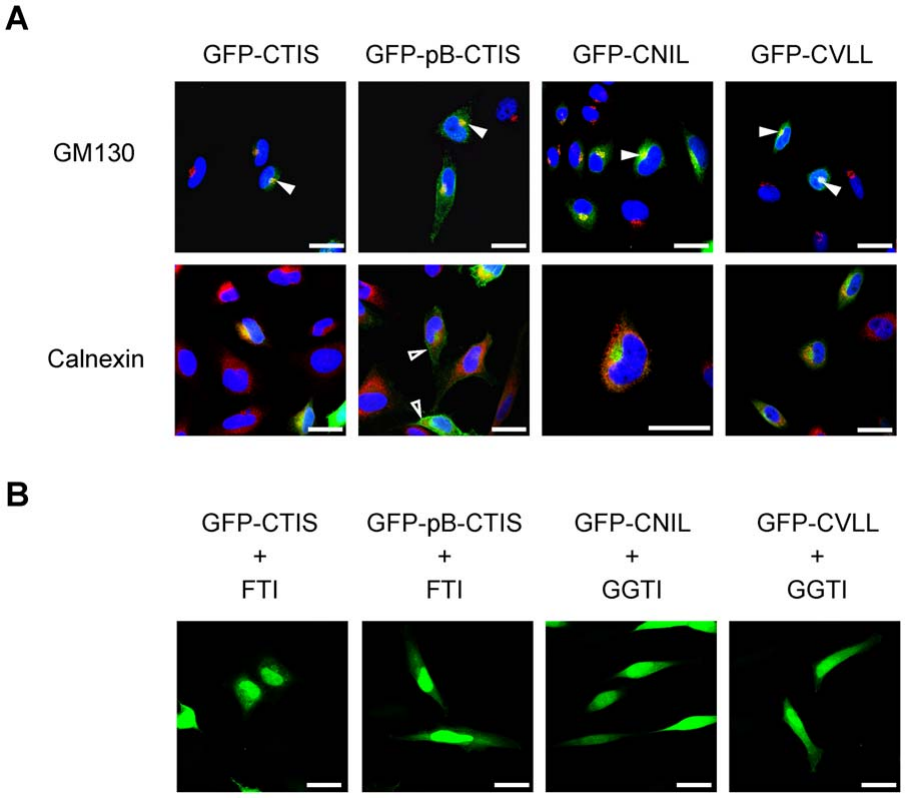
The CaaX motifs of GBPs direct GFP to the Golgi but not to the ER. (**A**) HeLa cells were transfected with Flag-GFP-CTIS, -CNIL, –CVLL or a construct containing the polybasic domain and the CaaX box of GBP-1 (GFP-pB-CTIS). Cells were stained with an antibody against the Golgi marker, GM130, and an anti-mouse-AlexaFluor 546 secondary antibody (upper panel) or against the ER marker, calnexin, (lower panel) using an anti-rabbit-AlexaFluor 546 secondary antibody. Nuclei were counterstained with DAPI. Co-localization with the Golgi marker GM130 is indicated by solid arrowheads. Localization at the plasma membrane is indicated by open arrowheads. (**B**) Prenylation inhibitors abrogate the localization of GFP-CTIS, GFP-CNIL, GFP-CVLL and GFP-pB-CTIS at the Golgi or the plasma membrane. HeLa cells were transiently transfected with Flag-GFP-CTIS, -pB-CTIS, -CNIL and -CVLL. Cells were treated with 10 µM FTI and 10 µM GGTI, as indicated. Scale bars = 25 µm.

Next, the influence of the different CaaX motifs alone on the localization was examined. To this end, the CaaX sequences of GBP-1, GBP-2 and GBP-5 (CTIS, CNIL and CVLL) were fused to the C-terminus of GFP and the intracellular localization of the respective proteins was analyzed (Fig. 3*A*). An additional construct containing the polybasic domain and the CaaX motif of GBP-1 was analyzed (Fig. 3*A*, GFP-pB-CTIS). All of the fusion proteins were present in the Golgi but not the ER, as shown by co-localization with GM130 (Fig. 3*A*, solid arrowheads) but not calnexin (Fig. 3*A*). GFP-pB-CTIS was observed at the plasma membrane in addition to the Golgi (Fig. 3*A*, open arrowheads). Treatment with the respective prenylation inhibitors abolished the localization of the fusion proteins at the Golgi (Fig. 3*B*). In conclusion, we showed that prenylation is necessary for the association of GBPs with membranes. However, the prenylation signals were not sufficient to determine alone the target compartment of GBP-1 and GBP-2.

### Homodimerization and GBP localization

**Figure 4:**
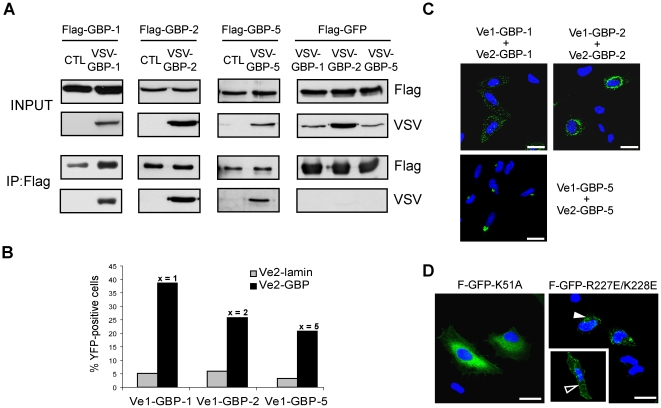
The *in vivo* homodimerization of GBPs differentially influences their intracellular localization. (**A**) GBP-1, GBP-2 and GBP-5 are able to homodimerize *in vivo*. HeLa cells were co-transfected with Flag-GBPs or Flag-GFP together with VSV-GBPs or empty control vector (CTL) as indicated. Protein extracts were immunoprecipitated with an anti-Flag affinity gel and subjected to western blot analysis. For each co-transfection, cell lysates (10 µg, INPUT) and IP eluates (1∶4 for Flag-detection and 3∶4 for VSV detection) were analyzed. (**B**) Quantitative evaluation of the bi-molecular fluorescence complementation assay. Venus1 plasmids encoding various GBPs were co-transfected with matching Venus2-GBP (black bars) or Venus2-lamin (gray bars) encoding plasmid into HeLa cells. The number of fluorescent cells was determined by FACS analysis after 72 h. (**C**) Bi-molecular fluorescence complementation assay. HeLa cells were transfected with the plasmids expressing GBP-1, -2 or -5 fused with Venus1 or VSV-Venus2, respectively. Nuclei were counterstained with DAPI. (**D**) HeLa cells were transfected with different mutants of GBP-1 fused to GFP. Flag-GFP-GBP-1-K51A is defective in nucleotide-dependent oligomerization. Flag-GFP-GBP-1-R227E/K228E is constitutively dimeric and localizes in vesicle-likes structures (solid arrowhead) or at the plasma membrane (open arrowhead). Nuclei were counterstained with DAPI. Scale bars = 25 µm.

Previous biochemical and structural *in vitro* studies revealed that GBP-1 can homodimerize in a GTP-dependent manner, which strongly stimulates its GTPase activity [30]. Accordingly, we investigated whether GBP-1, GBP-2 and GBP-5 were able to homodimerize *in vivo* using a co-immunoprecipitation approach. Flag-tagged and VSV-tagged GBPs were expressed in HeLa cells (Fig. 4*A*, INPUT). Immunoprecipitation of the Flag-tagged proteins resulted in the co-precipitation of the VSV-tagged counterparts (Fig. 4*A*, IP:Flag). Flag-GFP was used as a negative control and did not precipitate any VSV-GBP (Fig. 4*A*, right panel). Results were confirmed by reciprocal co-immunoprecipitation using an anti-VSV antibody (Figure S2 *A*). Homodimerization of GBP-1, GBP-2 and GBP-5 was confirmed using a yeast-two-hybrid assay (Text S1 and Fig. S3 *A*).

Next, the intracellular localization of homodimers of GBP-1, GBP-2 and GBP-5 was investigated using a bimolecular fluorescence complementation assay. In this assay, amino-terminal (Venus1) and carboxyl-terminal (Venus2) sub-fragments of the YFP mutant “Venus” can reconstitute and generate *de novo* fluorescence if fused to interacting proteins [31], [32], [33]. Venus1 and Venus2 were fused with the N-terminus of the three GBPs. Lamin C was used as control because it can dimerize but localizes to the nucleus and therefore should not contact the cytoplasmic GBPs [34]. In order to verify the specificity of the method, homodimerization of Venus-GBPs was quantitatively analyzed by flow cytometry. Significantly increased numbers of fluorescent cells were observed when Venus1- and Venus2-GBPs were co-transfected (Fig. 4*B*, black bars; GBP-1: 38%, GBP-2: 25.8% and GBP-5: 20.8%) as compared to co-transfection of each Venus1-GBP with Venus2-lamin (Fig. 4*B*, gray bars; <6%). The intracellular localization of the homodimers of GBP-1, GBP-2 and GBP-5 was then assessed using confocal microscopy (Fig. 4*C*). No signal was observed after co-expression of Venus1-lamin with VSV-Venus2-GBP-1, -2 or -5 (Fig. S3 *B*). The Venus interaction signal of GBP-1 was strictly granular indicating that homodimers of GBP-1 localize at vesicle-like structures (Fig. 4*C*, left panel). In addition, the signal partially concentrated at the plasma membrane. In the case of GBP-2, a strong Venus signal with a granular appearance was observed in the perinuclear region (Fig. 4*C*, right panel) but did not co-localize with the Golgi marker, GM130, or the ER marker, calnexin (Fig. S4 *A*). The GBP-5 homodimer localized at the Golgi (Fig. 4*C*, bottom panel). These results indicated that homodimers of GBP-1, GBP-2 and GBP-5 localize in specific compartments that only partly overlap with the general distribution of the proteins and suggested that homodimerization does influence the localization of GBPs.

In order to test the influence of prenylation on the localization of GBP homodimers, cells were treated with FTI or GGTI. Fluorescence complementation indicated that all three GBPs could still dimerize in the absence of prenylation. However the fluorescent signals were evenly distributed throughout the cytoplasm (Fig. S4 *B*), indicating that isoprenylation is necessary for membrane affinity of the dimers.

To characterize more precisely the effect of homodimerization on the localization of GBP-1, we studied the localization of two mutants known to be constitutively monomeric or dimeric, respectively (Fig. 4*D*). We first used the GTPase-defective mutant, K51A, which displays reduced nucleotide affinity and hydrolytic activity. This mutant lacks nucleotide-dependent oligomerization and thus is constitutively monomeric [35]. We found that the GFP-K51A fusion protein localized evenly throughout the cytoplasm without any granular accumulation (Fig. 4*D*). In addition, the K51A mutant was unable to form homodimers in the Venus fluorescence complementation assay (figure S5). In parallel, a constitutively dimeric mutant was generated (R227E/K228E) [36]. Interestingly, the R227E/K228E mutant was exclusively associated with vesicle-like structures and at the plasma membrane, where it co-localized with the actin cytoskeleton (Fig. 4*D*, open arrowhead, and S4*C*). These results indicated that GBP-1 localizes diffusely as a monomer and is associated with membranous structures as a dimer.

### Heterodimerization and GBP localization

**Figure 5:**
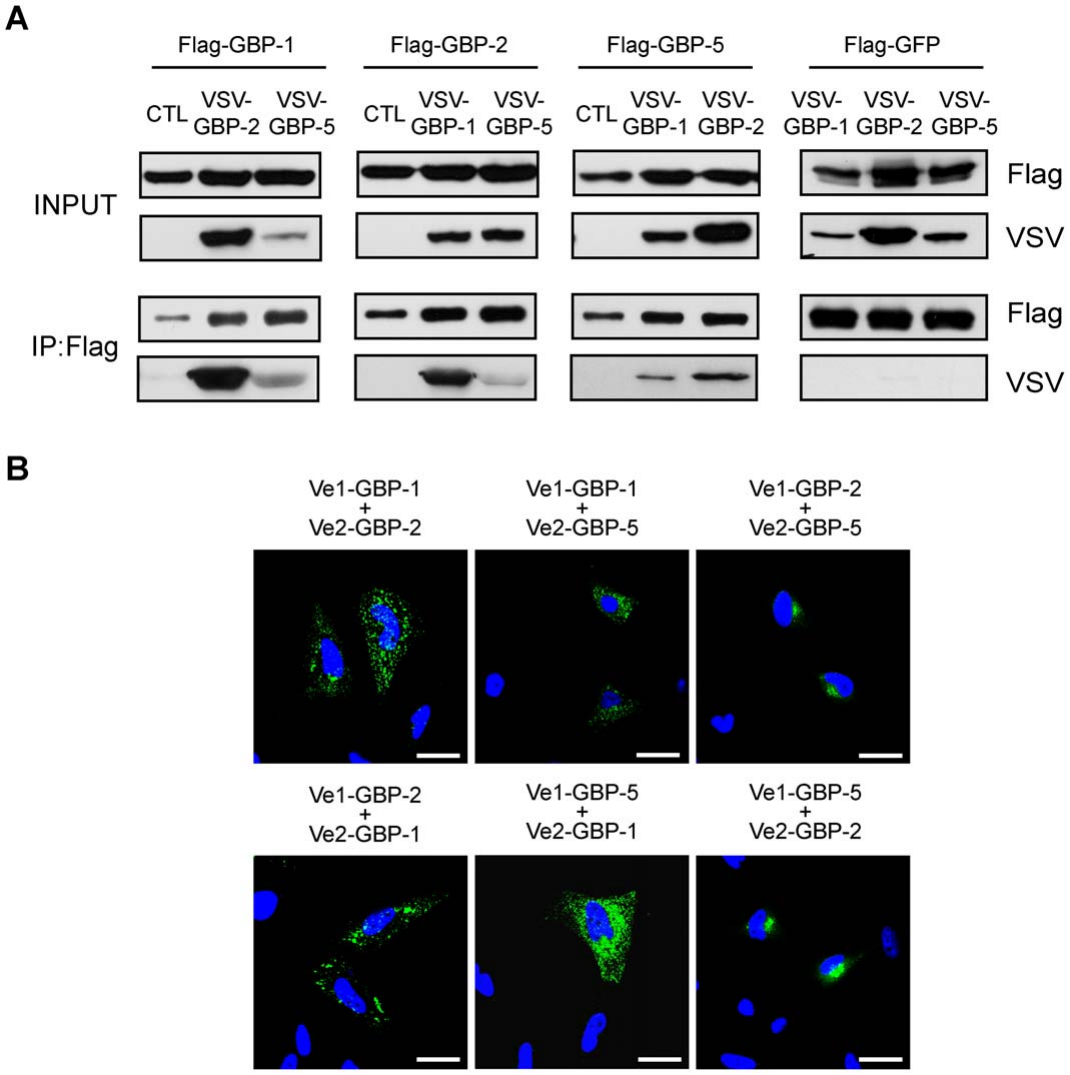
The intracellular localization of GBPs is regulated by heterodimerization in a hierarchical manner. (**A**) HeLa cells were co-transfected with Flag-GBPs, Flag-GFP and VSV-tagged GBPs or empty control vector (CTL) as indicated. Cell harvesting, Flag-immunoprecipitation and western blot analyses were performed as described in Fig. 3*A*. (**B**) HeLa cells were co-transfected with plasmids expressing Venus1-GBP and VSV-Venus2-GBP as indicated. Nuclei were counterstained with DAPI. Scale bars = 25 µm.

Lastly, it was investigated whether GBPs heterodimerized and whether this process influenced the subcellular localization of GBPs (Fig. 5). Co-immunoprecipitation with Flag- and VSV-tagged proteins showed that all three prenylated GBPs interact strongly with each other (Fig. 5*A*). Results were confirmed by reciprocal co-immunoprecipitation using an anti-VSV antibody (Figure S2 *B*). Fluorescence complementation confirmed at the single cell level that GBP-1, -2 and -5 can bind to each other (Fig. 5*B*). Co-expression of Venus-GBP-1 with Venus-GBP-2 or Venus-GBP-5 resulted in a granular fluorescence signal identical to the GBP-1 homodimer localization pattern (Fig. 5*B*). This indicated that GBP-1 directs both GBP-2 and GBP-5 to its intracellular compartment. The GBP-1 mutant K51A was used as negative control in the Venus fluorescence complementation assay and was unable to form heterodimers with wild-type GBP-1, GBP-2 and GBP-5 (figures S6). Co-expression of Venus-GBP-2 and Venus-GBP-5 resulted in the localization of the heterodimer at the Trans-Golgi (Fig. 5*B* and Fig. S7 *A*). Inhibition of GBP-1 farnesylation induced the localization of GBP-1/GBP-2 and GBP-1/GBP-5 heterodimers to the compartments of homodimeric GBP-2 and GBP-5, respectively (Fig. S7 *B*). Treatment with GGTI resulted in a diffuse localization pattern for GBP-2/GBP-5 heterodimers (Fig. S7 *B*). These results showed that GBPs heterodimers need to be prenylated in order to associate with membranous structures, similarly to the homodimers.

GFP-GBP-2 and GFP-GBP-5 were expressed in HeLa cells in presence or in absence of IFN-γ in order to investigate whether endogenous GBP-1 may also affect the localization of both proteins. After treatment with IFN-γ, GFP-GBP-2 displayed a vesicle-like pattern resembling the GBP-1 localization in 50% of the cells (Figure S8). In the case of GFP-GBP-5 also, treatment with IFN-γ induced a vesicle-like distribution but in a lower extend (∼20% of the cells) (Figure S8). These results indicated that endogenously interferon-induced GBP-1 can also redirect GBP-2 and GBP-5 to its vesicle-like compartment.

**Figure 6:**
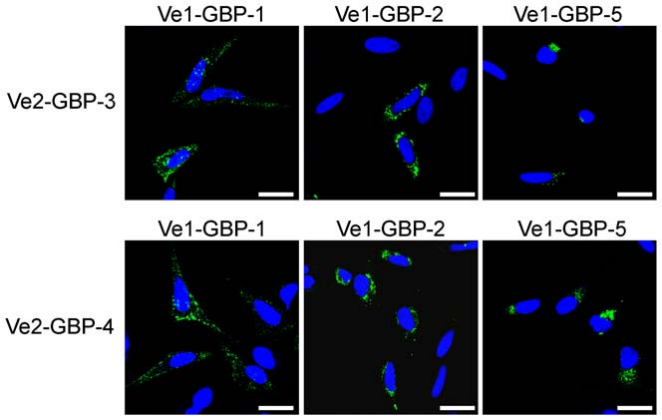
Prenylated GBPs recruit non-prenylated GBPs to their subcellular compartments. HeLa cells were co-transfected with plasmids expressing Venus1-GBP-1, -GBP-2 or -GBP-5 together with plasmids expressing GBP-3 or GBP-4 fused to VSV-Venus2. Nuclei were counterstained with DAPI. Scale bars = 25 µm.

Finally it was investigated whether the GBPs harbouring a CaaX box could bind to non-prenylated GBP-3 and -4, and if so, whether prenylated GBPs could influence the localization of GBP-3 and GBP-4. When the proteins are expressed singly, GBP-3 has been shown to localize in the cytoplasm in a diffuse manner whereas GBP-4 was evenly distributed throughout the nucleus and cytoplasm [11]. Both GBP-3 and GBP-4 formed heterodimers with GBP-1, GBP-2 and GBP-5, as shown by immunoprecipitation (Fig. S9 *A*). The heterodimers formed between GBP-3 or GBP-4 and GBP-1, -2 or -5 always localized in the compartment of the prenylated GBPs: with GBP-1 in a vesicle-like compartment, with GBP-2, around the nucleus and with GBP-5, at the Golgi (Fig. 6 and Fig. S9 *B*). These results indicated that GBPs can heterodimerize and influence the localization of each other in a hierarchical manner, with the prenylated GBPs being dominant over the non-prenylated GBPs.

## Discussion

In the present study, we showed for the first time that GBP-2 and GBP-5 are prenylated *in vivo* and we confirmed that GBP-1 is prenylated. In addition, we showed that prenylated GBPs are associated with cellular membranes. Interestingly, we found that all GBPs investigated here were able to form homodimers. In case of GBP-1, dimers exclusively displayed a vesicle-like pattern partially accumulating at the plasma membrane. Dimers of GBP-2 and GBP-5 were also associated with membranous compartments, however distinct from the GBP-1 compartment, such as the perinuclear membrane area in case of GBP-2 and the Golgi in case of GBP-5. Membrane association of the dimers is corroborated by the fact that stabilization of prenylated GBP-1, GBP-2 and GBP-5 in their transition state (which corresponds to a tetramer for GBP-1 and GBP-5) by treatment with aluminum fluoride leads to membrane association at the Golgi [11], [37]. In conclusion, our data indicated that association of GBPs with cellular membranes requires not only prenylation but also dimerization.

At this point we cannot exclude that the vesicle-like localization pattern of dimeric GBP-1 might correspond to protein aggregates. However, several lines of evidence support an association of the protein with membranous structures. Firstly, the observed vesicle-like structures were dependent on prenylation while oligomerization of GBP-1, observed *in vitro* at least to the level of tetramers, occurs independently of prenylation. Secondly, the presence of GBP-1 in vesicles is well in agreement with the fact that GBP-1 is secreted, whereas the formation of aggregates would rather support the retention of the protein in the cytoplasm [38]. Finally, it has been shown that farnesylated GBP-1 associates with liposomes in the active state of GTP hydrolysis [39].

We showed that a part of GBP-1 homodimers associates with the plasma membrane where they co-localize with the actin cytoskeleton. This finding suggests a role for human GBP-1 in signal transduction and cytoskeleton organization and is corroborated by the fact that GBP-1 is known to inhibit spreading of endothelial cells [18]. Recently, murine GBP-2, which is considered as the functional homolog of GBP-1, has been shown to bind and inactivate phosphoinositol 3-kinase, resulting in the inhibition of Rac activity and of cell spreading [40]. In addition, GBP-1 has been shown to associate with tight junctions under inflammatory conditions, however exclusively in intestinal epithelial cells [41]. Taken together, these data suggest a potential role of GBP-1 in vesicle and membrane biology.

The dimeric forms of all three prenylated GBPs were associated with different membranous compartments (GBP-1, vesicle-like, cytoplasmic or plasma membrane; GBP-2, perinuclear membranes; GBP-5, Golgi). In contrast, the isolated CaaX motifs of all GBPs uniformly targeted GFP to the Golgi. Association with additional targeting factors may be responsible for these different localizations. The action of chaperones has been evoked to explain the specific targeting of Ras, Rho, Rab or heterotrimeric α subunits GTPases to distinct organelles [42], [43], [44]. In particular, guanine dissociation inhibitor (GDI) proteins have been shown to bind and maintain Rho GTPases and Rab GTPases as soluble cytosolic proteins [42], [45], [46]. Dissociation from GDI, in the case of Rab GTPases through the action of GDI displacement factors (GDF), allows the GTPases to associate with membranes. Such proteins may also be involved in the intracellular trafficking of GBPs.

Dimerization functionally connects the regulation of membrane association with the GTPase cycle of GBPs in living cells. For example, GBP-1 hydrolyses GTP into GDP and GMP during two consecutive steps. First, GBP-1 forms dimers upon binding to GTP and is transiently present as a tetramer during the GDP-bound transition phase. Then, GDP is cleaved into GMP which results in dimer dissociation [21], [22], [47]. Accordingly, the mixed pattern of localization of wild-type GBP-1 might reflect the dynamic and transient association with membranes depending on the GTP hydrolysis cycle. In contrast to GBP-1, GBP-5 forms dimers in the nucleotide-free state [22]. Upon GTP binding, a larger complex is formed until GTP cleavage, when the protein complex dissociates and GBP-5 becomes again dimeric [22]. The constitutive dimeric state of GBP-5 corroborates the constitutive membrane association observed in this study. No biochemical data are available concerning the oligomeric state of GBP-2 during GTP hydrolysis.

Investigating putative co-operative effects between the different GBPs, we could show that all the GBPs included in this study have the ability to heterodimerize and that this results in a localization hierarchy within the GBPs. Specifically, GBP-1 recruited GBP-5 and GBP-2 into its own cellular compartment and GBP-5 repositioned GBP-2. In addition, GBP-1, GBP-2 and GBP-5 were able to redirect non-prenylated GBPs to their compartment in a prenylation-dependent manner. The same may apply for murine GBPs (mGBPs). It has been shown recently that after IFN-γ stimulation, all mGBPs are expressed and show a discrete vesicle-like structure inside the cytoplasm [48]. However, in the case of single ectopic expression, mGBP-1 is evenly distributed throughout the cytoplasm whereas mGBP-2 demonstrates a vesicle-like localization pattern [49]. Similarly, it has been shown that mouse IRG proteins, which also belong to the dynamin large GTPases family, are able to heterodimerize and to mutually regulate their subcellular localization [50].

Of note, a striking analogy in intracellular trafficking events has been described for heteromeric G receptor-coupled small GTPases. The three members of the family, the proteins Gα, Gβ and Gγ, can only reach the plasma membrane when they multimerize and are appropriately prenylated or acylated [51]. More specifically, dimers of Gβ and Gγ are formed in the cytoplasm and, upon prenylation of Gγ, the dimers reach the endoplasmic reticulum and the Golgi apparatus where they trimerize with the Gα subunit. Acylation of Gα at the Golgi then allows the G protein heterotrimer to be transported to the plasma membrane likely via the classic secretory pathway [51]. In the same way, GBPs may form heterodimers in the cytoplasm and may be prenylated and processed in the endoplasmic reticulum and the Golgi before they are targeted to their final membranous compartment.

Overall our data showed that the association of GBPs with membranes depends on prenylation and is regulated by dimerization. Moreover, GBPs are able to heterodimerize, which results in a re-localization of the dimers in a hierarchical manner. The vesicle-like localization pattern characteristic of GBP-1 homo- and heterodimers suggests a possible role in vesicle formation or trafficking, while the association with the actin cortex at the plasma membrane indicates a potential role in signal transduction or cytoskeleton organization.

## Materials and Methods

### Plasmids

The plasmids pMCV1.4 (−) and (+) were obtained from Mologen (Berlin, Germany). A Flag–tag sequence (abbreviation: F) and a VSV-tag sequence were cloned into the pMCV1.4(−) and (+) plasmids using EcoRV/EcoRI or NdeI/EcoRI restriction sites, respectively. The sequences of GFP and the various GBPs were inserted in-frame into pMCV1.4-Flag using the EcoRI restriction site (NCBI accession numbers: GBP-1, NM_002053.2; GBP-2, NM_004120.3; GBP-3: NM_018284.2; GBP-4: NM_052941.4; GBP-5, NM_052942.3). Plasmids expressing GFP-GBP fusion proteins were obtained by inserting the sequences of GBP-1, -2 or -5 into the pMCV1.4-Flag-GFP vector using SnaBI and SalI restriction sites. The plasmids, pMCV1.4-Flag-GFP-CTIS, -CNIL and –CVLL were generated by cloning the respective CaaX box coding sequence fused to a glycine linker (consisting of 4 glycines) between PstI and SalI restriction sites. The plasmids, pMCV1.4-Flag-GFP-GBP-1ΔCTIS, pMCV1.4-Flag-GFP-GBP-2ΔCNIL, pMCV1.4-Flag-GFP-GBP-5ΔCVLL and pMCV1.4-Flag-GFP-pB-CTIS were generated by site-directed mutagenesis (QuikChange II Mutagenesis kit, Stratagene, La Jolla, CA) using the primers GBP-1ΔCTIS forward (5′- CAGACGAAAATGAGACGACGAAAGGCATAAGTCGACGAATTCAAGC-3′), GBP-2ΔCNIL forward (5′-GCAAATCATTGGAGCCAATATAAGTCGACGAATTCAAGC-3′) and GBP-5ΔCVLL forward (5′-GGACTGTTAATAAACGATGATTCCATAAGTCGACG-3′) containing a SalI restriction site (underlined). The plasmids pCDNA3.1-Venus1 and pCDNA3.1-Venus2 were a kind gift from Dr. Stephen Michnick (University of Montreal, Montreal, Canada). The coding sequences of Venus1 and Venus2 were inserted between the EcoRI and EcoRV restriction sites of pMCV1.4 (+) and pMCV1.4-VSV, respectively. The coding sequences of lamin C, GBP-1, GBP-1 K51A, GBP-2, GBP-3, GBP-4 and GBP-5 were then inserted into pMCV1.4-Venus1 and pMCV1.4-VSV-Venus2 at the C-terminus of the Venus sequence using the EcoRV and XhoI restriction sites.

### Cell culture and transfections

HeLa cells were purchased from ATCC and cultured in DMEM supplemented with 10% (v/v) fetal bovine serum (FBS) from PAA (Pasching, Austria). Transfections were performed using the calcium phosphate method [52].

### Immunofluorescence analysis

For immunofluorescence and bimolecular fluorescence complementation assay, HeLa cells were seeded in Lab-Tek chamberslides (Nunc™, Thermo Fisher Scientific, Bonn, Germany). One microgram of plasmid was transfected per well. When indicated, 100 U/ml of IFN-γ (Roche Applied Science, Mannheim, Germany), 10 µM of GGTase-I inhibitor (GGTI-2147) or 10 µM of FTase inhibitor (L744,832) (both from Calbiochem™, Merck, Darmstadt, Germany) were added to the cells 8 h after transfection. Twenty-four hours after transfection, cells were fixed in 10% buffered formalin (Sigma-Aldrich) and permeabilized in 0.1% Triton X100 (Sigma-Aldrich). The following antibodies were used for immunofluorescence staining: a rabbit anti-Flag-tag antibody (diluted 1∶500) (ABR, Thermo Fisher Scientific), a mouse monoclonal anti-VSV (diluted 1∶5,000) (Sigma-Aldrich, Schnelldorf, Germany), a mouse anti-GM130 antibody (clone 35, diluted 1∶1,000) (BD Biosciences, San Jose, CA), a rabbit anti-calnexin antibody (diluted 1∶500) (Santa Cruz, Santa Cruz, CA) or a rabbit anti-actin antibody (diluted 1∶100) (Sigma-Aldrich). Antibodies were incubated overnight at 4°C in 1× TBS with 5% normal goat serum (Dianova, Hamburg, Germany). After washing, cells were incubated for 1 h at room temperature with the following secondary antibodies (diluted 1∶500): AlexaFluor 546-conjugated goat anti-rabbit IgG, AlexaFluor 546-conjugated goat anti-mouse IgG or AlexaFluor 488-conjugated goat anti-rabbit IgG (Invitrogen/Molecular Probes, Darmstadt, Germany). Nuclei were counterstained with DAPI (1∶5,000 in water, Invitrogen/Molecular probes) for 10 min at room temperature. Coverslips were mounted in fluorescence mounting medium (Dako, Hamburg, Germany). Fluorescence was visualized using the confocal microscope, TCS SP5 (Leica Microsystems, Wetzlar, Germany) using a 63× magnification. Pictures were obtained using the LAS AF software (Leica Microsystems) and the Adobe® Photoshop® CS2 software. All images presented are single sections in the z-plane (airy unit = 1) and are representative of at least 80% of the transfected cells.

### Subcellular fractionation

HeLa cells were seeded in 10-cm dishes and transfected after 24 h with 10 µg of the plasmids, pMCV1.4-Flag-GBP-1, -2, -3, -4 or -5 and pMCV1.4-Flag-GBP-1, -2 or -5ΔCaaX using the calcium phosphate method. Twenty-four hours after transfection, 2.5×10^6^ cells were harvested and cytoplasmic, membranous or nuclear fractions were purified using the Qproteome fractionation kit (Qiagen, Hilden, Germany) according to the manufacturer's recommendations.

### Immunoprecipitation (IP)

HeLa cells were seeded in culture dishes (Nunc™) and transfected 24 h later with 10 to 15 µg of DNA as described above. Cells were harvested 48 h after transfection with a sterile cell scraper (Nunc) in 1.5 ml of ice-cold IP-lysis buffer (20 mM Tris-HCl pH 7.5, 150 mM NaCl, 5 mM MgCl_2_, 1% Igepal and EDTA-free inhibitor mix: Roche Applied Science). For immunoprecipitation, 750 µg of protein were incubated with 10 µl of anti-Flag affinity gel M2 (Sigma-Aldrich) or 5 µl of rabbit anti-VSV antibody bound to 50 µl of protein A-agarose beads (both Sigma-Aldrich) overnight at 4°C. Samples were washed with IP-wash buffer (20 mM Tris-HCl pH 7.5, 150 mM NaCl, 5 mM MgCl_2_, 0.1% Igepal). Immunoprecipitated proteins were eluted from the beads by addition of 30 µl of Laemmli buffer [53] and boiled for 10 min. The protein concentration of IP-lysates was determined using the DC assay (Bio-Rad Laboratories, Munich, Germany). IP-lysates (10 µg) were resuspended using 2× Laemmli buffer and subsequently subjected to western blot analysis.

### Western blot analysis

Ten micrograms of IP-lysates, 8 to 25 µl of IP-eluates or 15 µl of fractionated samples were separated by electrophoresis on 10% SDS-PAGE gels and analyzed by western blot as described previously [16]. The following primary antibodies were used: mouse monoclonal anti-Flag M2 (1∶10,000), mouse monoclonal anti-VSV-G (1∶40,000), rabbit polyclonal anti-Flag (1∶400), rabbit polyclonal anti-VSV-G (1∶1,000) (each from Sigma-Aldrich), mouse anti-GAPDH (1∶60,000, Chemicon), goat anti-lamin A/C (1∶1,000, Santa Cruz) and mouse anti-pan-cadherin (1∶2,000, Abcam). A donkey anti-rabbit IgG antibody coupled to horseradish peroxidase (HRP) (GE Healthcare, Munich, Germany), a donkey anti-goat-HRP (GE Healthcare) and a sheep anti-mouse-HRP antibody (Dako) were used as the secondary antibodies at the dilution of 1∶5,000. Protein detection was performed using the enhanced chemiluminescence western blot detection system (ECL, GE Heathcare) and Rx-films (Fuji, Tokyo, Japan).

### Radiolabelling with [^3^H]-mevalonolactone ([^3^H]-MVA)

HeLa cells were seeded in 6-wells plates and transfected after 24 h with 5 µg of the plasmids, pMCV1.4-Flag-GBP-1, -2 or -5 and pMCV1.4-Flag-GBP-1, -2 or -5ΔCaaX. Cells were incubated for 6 h and then rinsed two times with 1× PBS. Fresh medium (DMEM+10%FCS) supplemented with 30 µM of Lovastatin (Sigma-Aldrich) was added. After 3 h, medium was renewed and 200 µCi of [^3^H]-MVA (Biotrend Chemikalien, Cologne, Germany) was added per well. Cells were incubated for further 20 h. Cells were lysed and proteins were immunoprecipitated using an anti-Flag monoclonal antibody (M2, Sigma-Aldrich) as described above. One fifth of the immunoprecipitatted was used for quantitative determination of the radioactivity in 5 ml of Ultima Gold (Perkin-Elmer, Rotgau, Germany) with a liquid scintillator analyzer (Perkin-Elmer). The remaining 4/5 of the immunoprecipitates was separated by electrophoresis on a 10% SDS-PAGE gel. The gel was stained for 3 h with Coomassie blue staining solution (0,025% Coomassie brilliant blue R250, 40% methanol and 7% acetic acid) and destained for 1 h with 40% methanol and 7% acetic acid. The gel was then equilibrated for 30 min in 4% glycerol (Sigma-Aldrich), incubated for 30 min in Amplify® solution (GE Healthcare), dried overnight using a drying frame and a cellulose sheet (Invitrogen), and exposed for 3 weeks with a pre-flashed autoradiography film (GE Healthcare) at −70°C.

### FACS analysis

In order to analyze homomeric interactions by flow cytometry, 1 µg (GBP1 and −2) or 2 µg (GBP5) of Venus1 and Venus2 constructs were co-transfected into HeLa cells in 6-well plates as described above. Cells were detached after 72 h by standard trypsin/EDTA treatment, collected by centrifugation at 500×*g* (4°C, 5 min), washed twice in PBS supplemented with 5% FCS (4°C), and resuspended in PBS plus 5% FCS (4°C) for subsequent fluorescent cell enumeration using the FL1 channel (530/30 nm filter) of a FACSCalibur cytometer (Becton Dickinson, Franklin Lakes, NJ, USA).

### In silico analyses

The analysis of potential prenylation sites was performed using the prenylation prediction suite PrePS [54]. The search for palmitoylation or myristoylation signals was performed with the NMT [55] and the CSS-Palm [56] online applications.

## Supporting Information

Text S1 (0.02 MB DOC)

Figure S1 Controls for the subcellular localization of GBPs. (A) Subcellular distribution of GBP-3 and GBP-4. HeLa cells expressing Flag-GBP-3 or -4 were fractionated into cytosolic, membranous and nuclear fractions which were analyzed by western blot. GAPDH was used as a marker of the cytosolic fraction, lamin A/C as a marker of the nuclear fraction and cadherin as a marker for the membrane fraction. (B) Localization of Flag-GBP-1, Flag-GBP-2 and Flag-GBP-5. HeLa cells were transiently transfected with plasmids expressing Flag-GBP-1, Flag-GBP-2 and Flag-GBP-5. Cells were stained with an anti-Flag antibody and an anti-rabbit-AlexaFluor 546 secondary antibody, and nuclei were counterstained with DAPI. The presence of GBP-2 in the nucleus is indicated by an asterisk. (C) Controls for prenylation inhibition. HeLa cells were transiently transfected with Flag-GFP-GBP-1, Flag-GFP-GBP-2 and Flag-GFP-GBP-5. Cells were treated with 10 µM FTI and 10 µM GGTI, as indicated. Nuclei were counterstained with DAPI. Scale bars = 25 µm.
(5.42 MB TIF)

Figure S2 Reciprocal co-immunoprecipitations. HeLa cells were co-transfected with Flag-GBPs or Flag-GFP together with VSV-GBPs or empty control vector (CTL) as indicated. Protein extracts were immunoprecipitated with an anti-VSV antibody bound to protein A-agarose and subjected to western blot analysis. For each co-transfection, cell lysates (10 µg, INPUT) and IP eluates (1∶2 for VSV detection and 1∶2 for Flag detection) were analyzed.
(3.67 MB TIF)

Figure S3 In vivo homodimerization of GBPs.(A) Yeast two hybrid analysis of homophilic GBP interactions. Two haploid yeast strains expressing test proteins fused to the DNA-binding (DA) or activation domain (AD) of the transcription factor Gal4, were mated to diploid yeast cells and streaked out on selection plates lacking tryptophan and leucine (diploid growth control) or on plates additionally lacking histidine and adenine (interaction test plates). Interactions between test proteins enable yeast growth on interaction test plates. Test proteins were Gal4 DA-GBP1 (G1), -GBP2 (G2), -GBP3 (G3), -GBP4 (G4), -GBP5 (G5), and Gal4-AD (Ctrl). (B) Controls for bi-molecular fluorescence complementation assay. HeLa cells were co-transfected with plasmids expressing Venus1-lamin and VSV-Venus2-lamin, -GBP-1, -GBP-2 or -GBP-5. VSV-Venus2 fusion proteins were stained with an anti-VSV antibody and an anti-mouse-AlexaFluor 546 secondary antibody, and nuclei were counterstained with DAPI. The positive control (Venus1-lamin × Venus2-lamin) confirmed that lamin C can dimerize and forms nucleoplasmic foci after overexpression (62). The negative controls showed that lamin can not bind to GBPs. Scale bars = 25 µm.
(9.05 MB TIF)

Figure S4 Prenylation-dependent localization of GBP homodimers.(A) Dimers of GBP-2 do not co-localize with the Golgi or the ER. HeLa cells were co-transfected with plasmids expressing Venus-1-GBP-2 and VSV-Venus2-GBP-2. Cells were stained with an anti-GM130 antibody and an anti-mouse-AlexaFluor 546 secondary antibody (left) or an anti-calnexin antibody and an anti-rabbit-AlexaFluor 546 secondary antibody (right). Nuclei were counterstained with DAPI. (B) Homodimerization of GBP-1, GBP-2 and GBP-5 is prenylation-dependent. HeLa cells were pairwise co-transfected with plasmids expressing Venus-1-GBP-1, -GBP-2 or -GBP-5 together with VSV-Venus2-GBP-1, -GBP-2 or -GBP-5, respectively. Cells were treated with 10 µM GGTI or 10 µM FTI as indicated. (C) GBP-1 R227E/K228E colocalizes with the actin cytoskeleton. HeLa cells were transfected with Flag-GFP-GBP-1-R227E/K228E. Cells were stained with an anti-actin antibody and an anti-rabbit-AlexaFluor 546 secondary antibody, and nuclei were counterstained with DAPI. Co-localization is indicated by solid arrowheads. Scale bars = 25 µm.
(7.76 MB TIF)

Figure S5 The GBP-1 mutant K51A is unable to form homo- or heterodimers. HeLa cells were co-transfected with plasmids expressing Venus1-GBP-1 K51A and VSV-Venus2-GBP-1 K51A, -GBP-1, -GBP-2 or -GBP-5. VSV-Venus2 fusion proteins were stained with an anti-VSV antibody and an anti-mouse-AlexaFluor 546 secondary antibody, and nuclei were counterstained with DAPI. Scale bars = 25 Âµm.
(9.66 MB TIF)

Figure S6 Reciprocal fluorescence complementation assay. HeLa cells were co-transfected with plasmids expressing Venus 1-GBP-1, -GBP-2 or -GBP-5 and VSV-Venus2-GBP-1 K51A. VSV-Venus2 fusion proteins were stained with an anti-VSV antibody and an anti-mouse-AlexaFluor 546 secondary antibody, and nuclei were counterstained with DAPI. Scale bars = 25 µm.
(8.71 MB TIF)

Figure S7 Prenylation-dependent localization of GBP heterodimers.(A) Heterodimers of GBP-2 and GBP-5 localize at the Golgi. HeLa cells were co-transfected with plasmids expressing Venus1-GBP-2 and VSV-Venus2-GBP-5. Cells were stained with an anti-GM130 antibody and an anti-mouse-AlexaFluor 546 secondary antibody, and nuclei were counterstained with DAPI. (B) Prenylation is necessary for membrane association of heterodimers of GBPs. HeLa cells were co-transfected with the expression plasmids of Venus1-GBP-2 or -5 and VSV-Venus2-GBP-1. The reciprocal experiments were performed with Venus-1-GBP-2 and Venus1-GBP-5. VSV-Venus2 fusion proteins were stained with an anti-VSV antibody and an anti-mouse-AlexaFluor 546 secondary antibody, and nuclei were counterstained with DAPI. Cells were treated with 10 µM GGTI or 10 µM FTI when indicated. Scale bars = 25 µm.
(5.06 MB TIF)

Figure S8 Redistribution of ectopically expressed GBPs in presence of IFN-γ. HeLa cells were transiently transfected with Flag-GFP-GBP-1, Flag-GFP-GBP-2 and Flag-GFP-GBP-5. Cells were treated with 100 U/ml of IFN-γ as indicated. Nuclei were counterstained with DAPI. Scale bars = 25 µm.
(3.95 MB TIF)

Figure S9 Homo- and heterodimerization of GBP-3 and GBP-4. (A) GBP-3 and GBP-4 are able to homodimerize and to heterodimerize. HeLa cells were co-transfected with Flag-GBPs together with VSV-GBPs, as indicated. Protein extracts were immunoprecipitated with an anti-Flag affinity gel and subjected to western blot analysis. For each co-transfection, cell lysates (10 µg, INPUT) and IP eluates (1∶4 for Flag-detection and 3∶4 for VSV detection) were analyzed. (B) GBP-3 and GBP-4 do not dimerize with lamin C. HeLa cells were co-transfected with plasmids expressing Venus1-lamin and VSV-Venus2-GBP-3 or -GBP-4. Nuclei were counterstained with DAPI. Scale bars = 25 µm.
(3.66 MB TIF)
