# Supplementary material for: Intracellular Trafficking of Guanylate-Binding Proteins Is Regulated by Heterodimerization in a Hierarchical Manner
Source: PLoS One. 2010 Dec 7;5(12):e14246. doi: 10.1371/journal.pone.0014246 (PMC2998424; doi:10.1371/journal.pone.0014246)
Supplement: Text S1 — (0.02 MB DOC) [file pone.0014246.s001.doc]

***Text S1: Supplementary material and methods***

**Yeast two hybrid assays.** Yeast culture, transformation and two hybrid assays were performed according to the yeast protocol handbook and the Matchmaker 3 manual (Clontech, Heidelberg, Germany). Briefly, AH109 yeast cells were transformed with pGBKT7- and Y187 yeast cells with pGADT7-constructs by the lithium acetate method. Transformants were selected on selection medium plates lacking tryptophan or leucine. Mating of two haploid yeast cells of different mating types to create a diploid yeast cell was used to assess the interaction capability of two test proteins. Haploid AH109 (mating type a) yeast expressing the bait protein and haploid Y187 (mating type α) yeast expressing a potential interaction partner (prey) were inoculated in 2x YPDA medium and incubated over night at 30°C with 130 rpm shaking. Thereafter, 1:10 dilutions in 0.5x YPDA were plated on diploid selection plates lacking trytophan and leucine (DDO, diploid growth control plates) and interaction selection plates without trytophan, leucine, histidine and adenine (QDO, interaction test plates). After incubation for 3 days at 30°C colonies on DDO plates demonstrated successful mating to diploid yeast. Growth of colonies on corresponding QDO plates monitored interaction of the two test proteins.
