# Supplementary figures and images for: Intracellular Trafficking of Guanylate-Binding Proteins Is Regulated by Heterodimerization in a Hierarchical Manner
Source: PLoS One. 2010 Dec 7;5(12):e14246. doi: 10.1371/journal.pone.0014246 (PMC2998424; doi:10.1371/journal.pone.0014246)

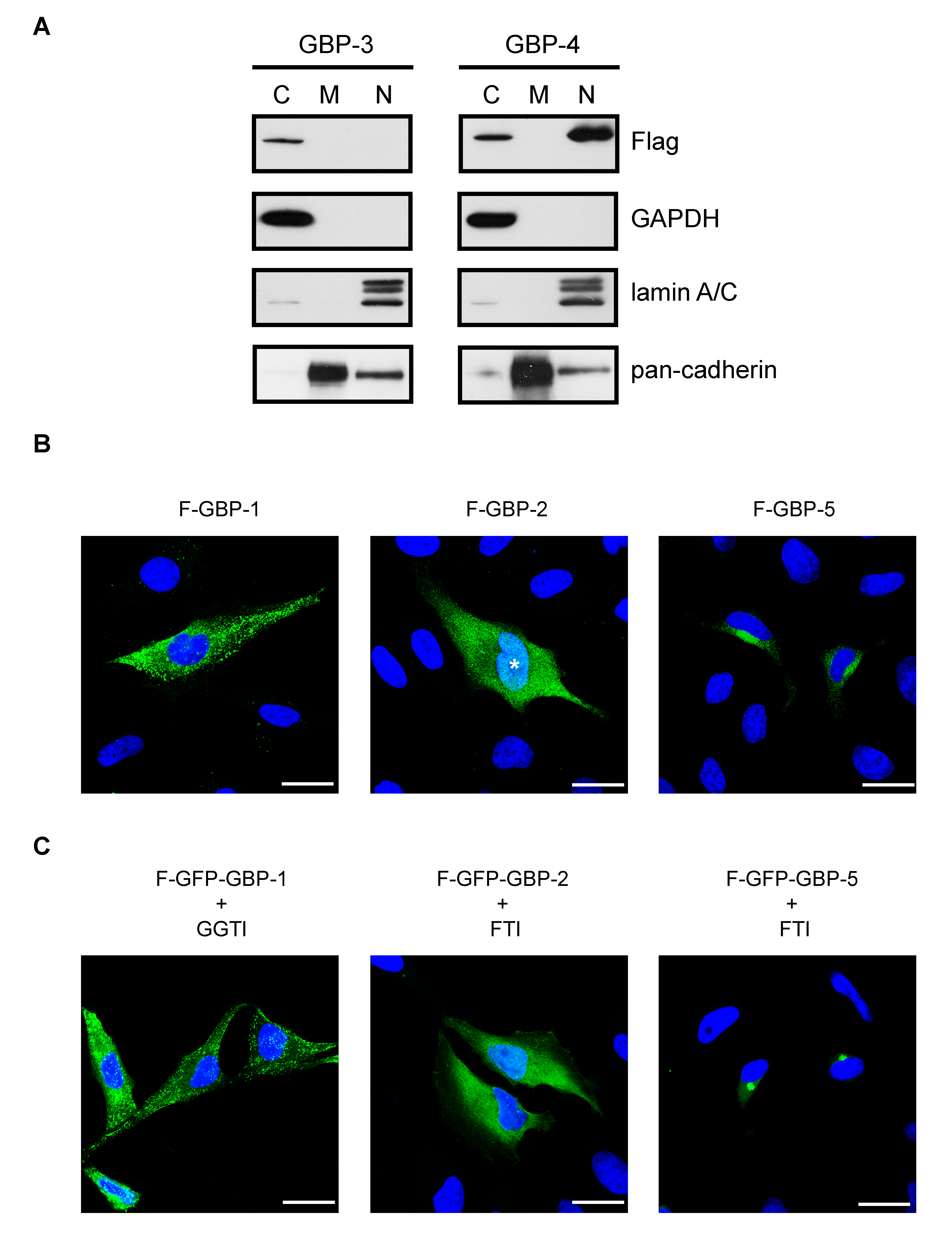

Supplement: Figure S1 — Controls for the subcellular localization of GBPs. (A) Subcellular distribution of GBP-3 and GBP-4. HeLa cells expressing Flag-GBP-3 or -4 were fractionated into cytosolic, membranous and nuclear fractions which were analyzed by western blot. GAPDH was used as a marker of the cytosolic fraction, lamin A/C as a marker of the nuclear fraction and cadherin as a marker for the membrane fraction. (B) Localization of Flag-GBP-1, Flag-GBP-2 and Flag-GBP-5. HeLa cells were transiently transfected with plasmids expressing Flag-GBP-1, Flag-GBP-2 and Flag-GBP-5. Cells were stained with an anti-Flag antibody and an anti-rabbit-AlexaFluor 546 secondary antibody, and nuclei were counterstained with DAPI. The presence of GBP-2 in the nucleus is indicated by an asterisk. (C) Controls for prenylation inhibition. HeLa cells were transiently transfected with Flag-GFP-GBP-1, Flag-GFP-GBP-2 and Flag-GFP-GBP-5. Cells were treated with 10 µM FTI and 10 µM GGTI, as indicated. Nuclei were counterstained with DAPI. Scale bars = 25 µm. (5.42 MB TIF) [file pone.0014246.s002.tif]

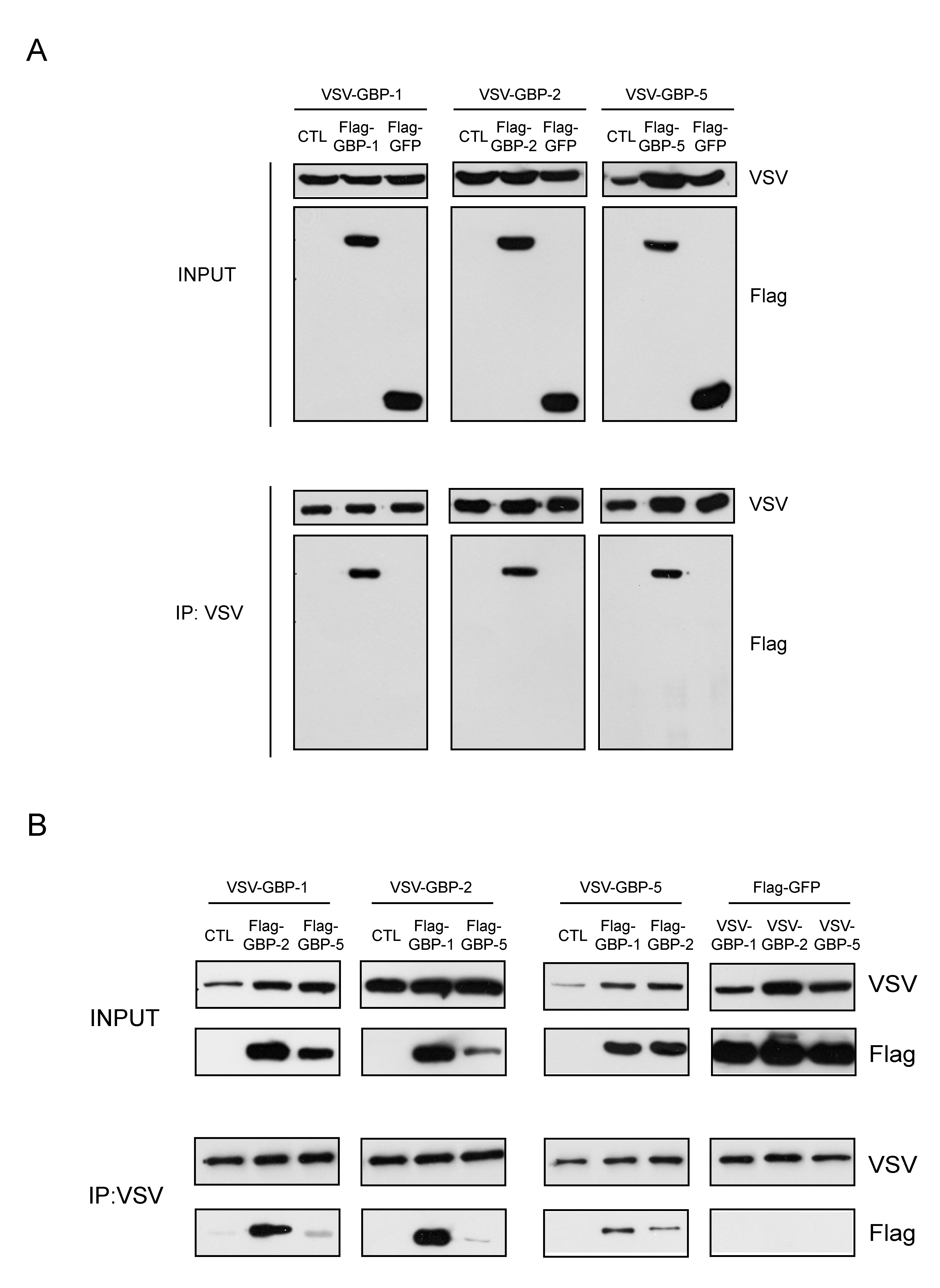

Supplement: Figure S2 — Reciprocal co-immunoprecipitations. HeLa cells were co-transfected with Flag-GBPs or Flag-GFP together with VSV-GBPs or empty control vector (CTL) as indicated. Protein extracts were immunoprecipitated with an anti-VSV antibody bound to protein A-agarose and subjected to western blot analysis. For each co-transfection, cell lysates (10 µg, INPUT) and IP eluates (1∶2 for VSV detection and 1∶2 for Flag detection) were analyzed. (3.67 MB TIF) [file pone.0014246.s003.tif]

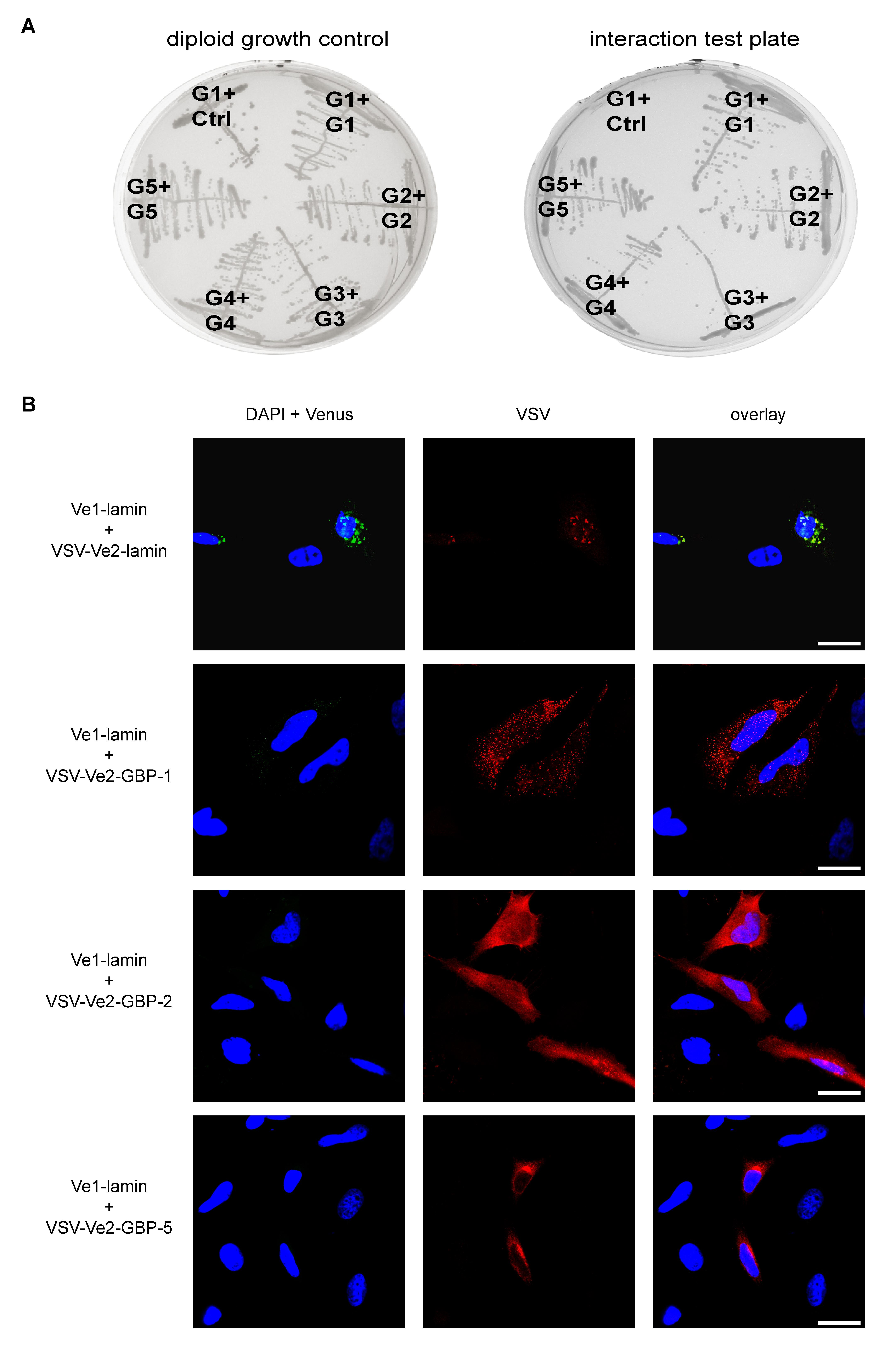

Supplement: Figure S3 — In vivo homodimerization of GBPs.(A) Yeast two hybrid analysis of homophilic GBP interactions. Two haploid yeast strains expressing test proteins fused to the DNA-binding (DA) or activation domain (AD) of the transcription factor Gal4, were mated to diploid yeast cells and streaked out on selection plates lacking tryptophan and leucine (diploid growth control) or on plates additionally lacking histidine and adenine (interaction test plates). Interactions between test proteins enable yeast growth on interaction test plates. Test proteins were Gal4 DA-GBP1 (G1), -GBP2 (G2), -GBP3 (G3), -GBP4 (G4), -GBP5 (G5), and Gal4-AD (Ctrl). (B) Controls for bi-molecular fluorescence complementation assay. HeLa cells were co-transfected with plasmids expressing Venus1-lamin and VSV-Venus2-lamin, -GBP-1, -GBP-2 or -GBP-5. VSV-Venus2 fusion proteins were stained with an anti-VSV antibody and an anti-mouse-AlexaFluor 546 secondary antibody, and nuclei were counterstained with DAPI. The positive control (Venus1-lamin × Venus2-lamin) confirmed that lamin C can dimerize and forms nucleoplasmic foci after overexpression (62). The negative controls showed that lamin can not bind to GBPs. Scale bars = 25 µm. (9.05 MB TIF) [file pone.0014246.s004.tif]

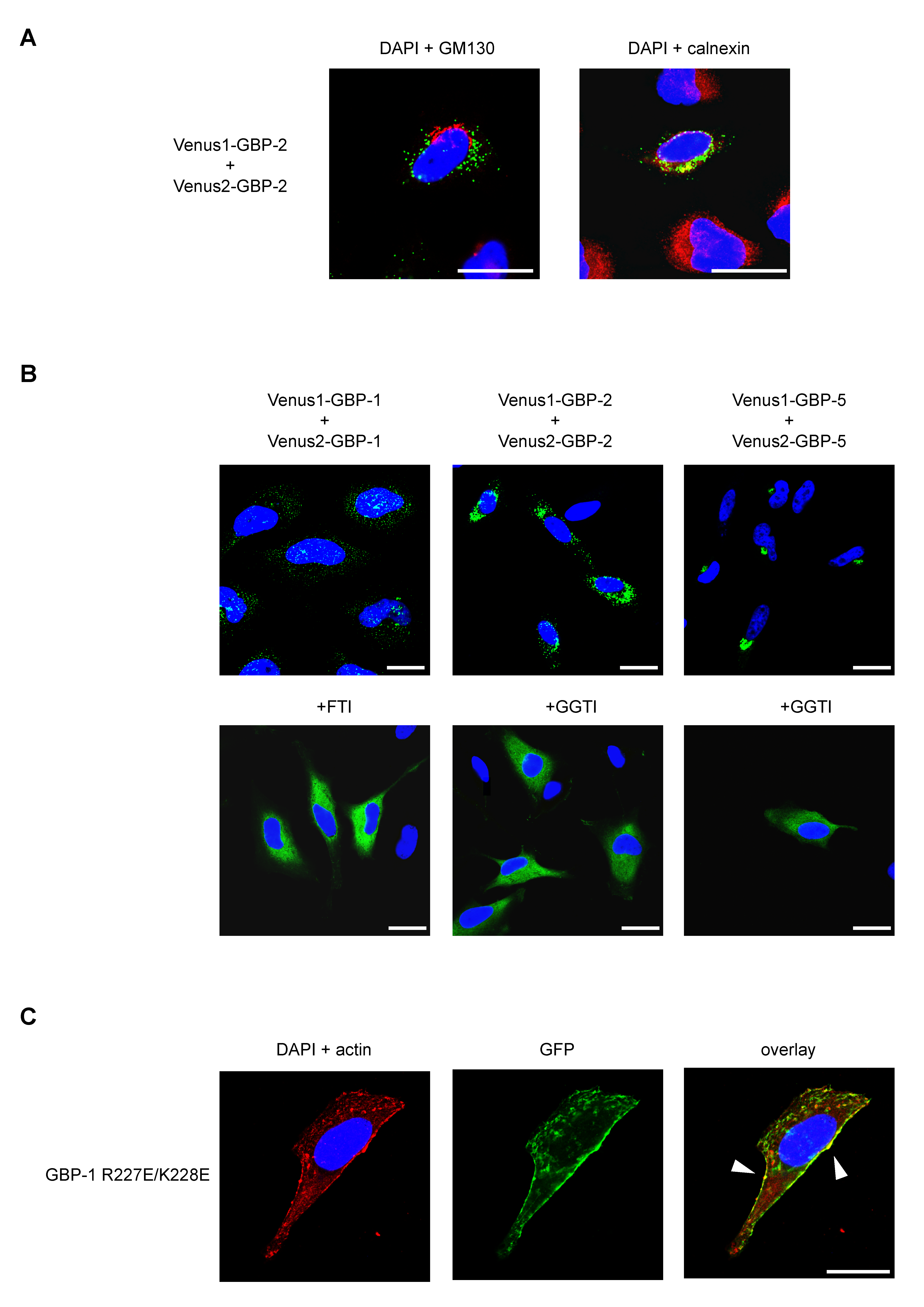

Supplement: Figure S4 — Prenylation-dependent localization of GBP homodimers.(A) Dimers of GBP-2 do not co-localize with the Golgi or the ER. HeLa cells were co-transfected with plasmids expressing Venus-1-GBP-2 and VSV-Venus2-GBP-2. Cells were stained with an anti-GM130 antibody and an anti-mouse-AlexaFluor 546 secondary antibody (left) or an anti-calnexin antibody and an anti-rabbit-AlexaFluor 546 secondary antibody (right). Nuclei were counterstained with DAPI. (B) Homodimerization of GBP-1, GBP-2 and GBP-5 is prenylation-dependent. HeLa cells were pairwise co-transfected with plasmids expressing Venus-1-GBP-1, -GBP-2 or -GBP-5 together with VSV-Venus2-GBP-1, -GBP-2 or -GBP-5, respectively. Cells were treated with 10 µM GGTI or 10 µM FTI as indicated. (C) GBP-1 R227E/K228E colocalizes with the actin cytoskeleton. HeLa cells were transfected with Flag-GFP-GBP-1-R227E/K228E. Cells were stained with an anti-actin antibody and an anti-rabbit-AlexaFluor 546 secondary antibody, and nuclei were counterstained with DAPI. Co-localization is indicated by solid arrowheads. Scale bars = 25 µm. (7.76 MB TIF) [file pone.0014246.s005.tif]

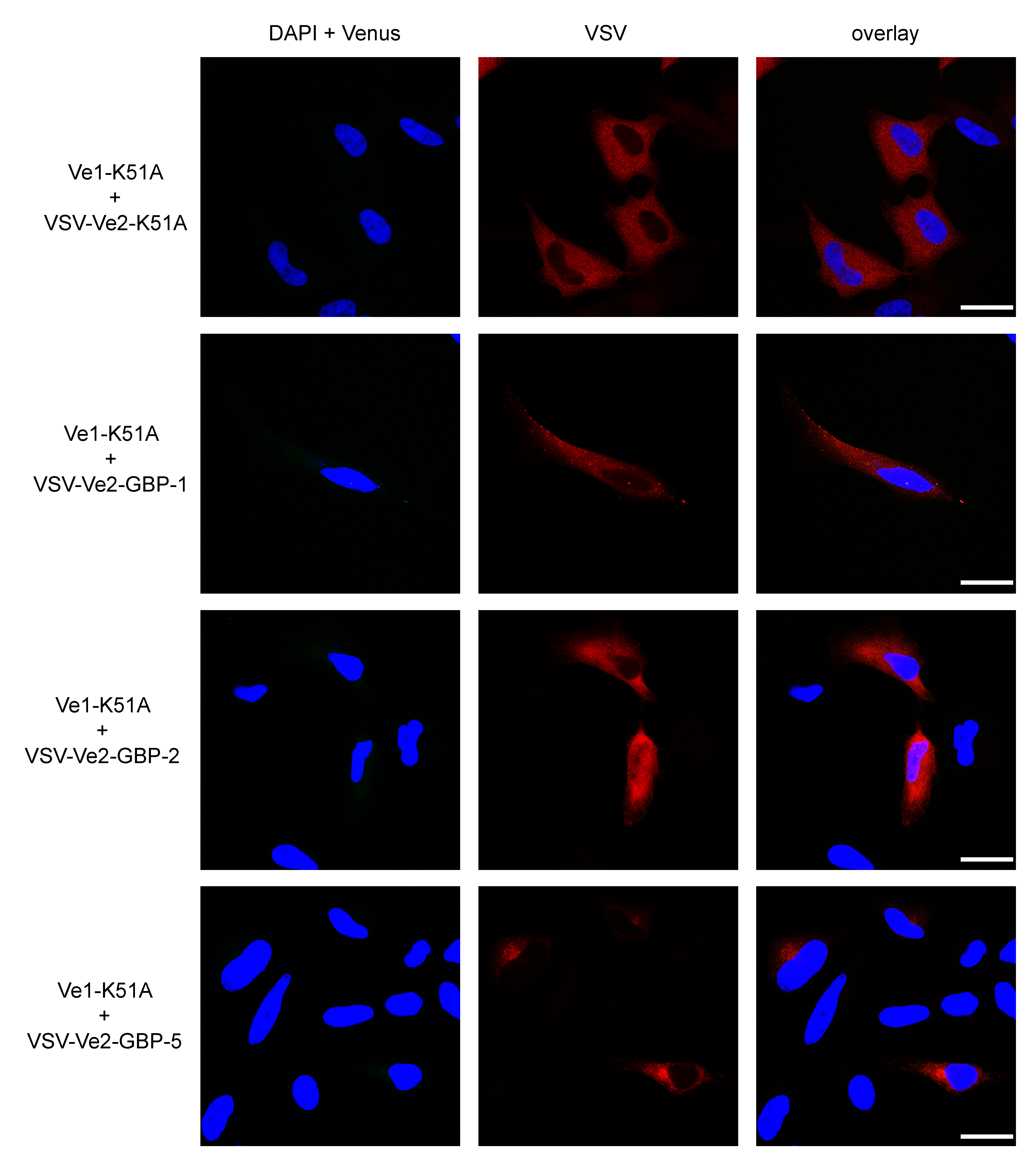

Supplement: Figure S5 — The GBP-1 mutant K51A is unable to form homo- or heterodimers. HeLa cells were co-transfected with plasmids expressing Venus1-GBP-1 K51A and VSV-Venus2-GBP-1 K51A, -GBP-1, -GBP-2 or -GBP-5. VSV-Venus2 fusion proteins were stained with an anti-VSV antibody and an anti-mouse-AlexaFluor 546 secondary antibody, and nuclei were counterstained with DAPI. Scale bars = 25 Âµm. (9.66 MB TIF) [file pone.0014246.s006.tif]

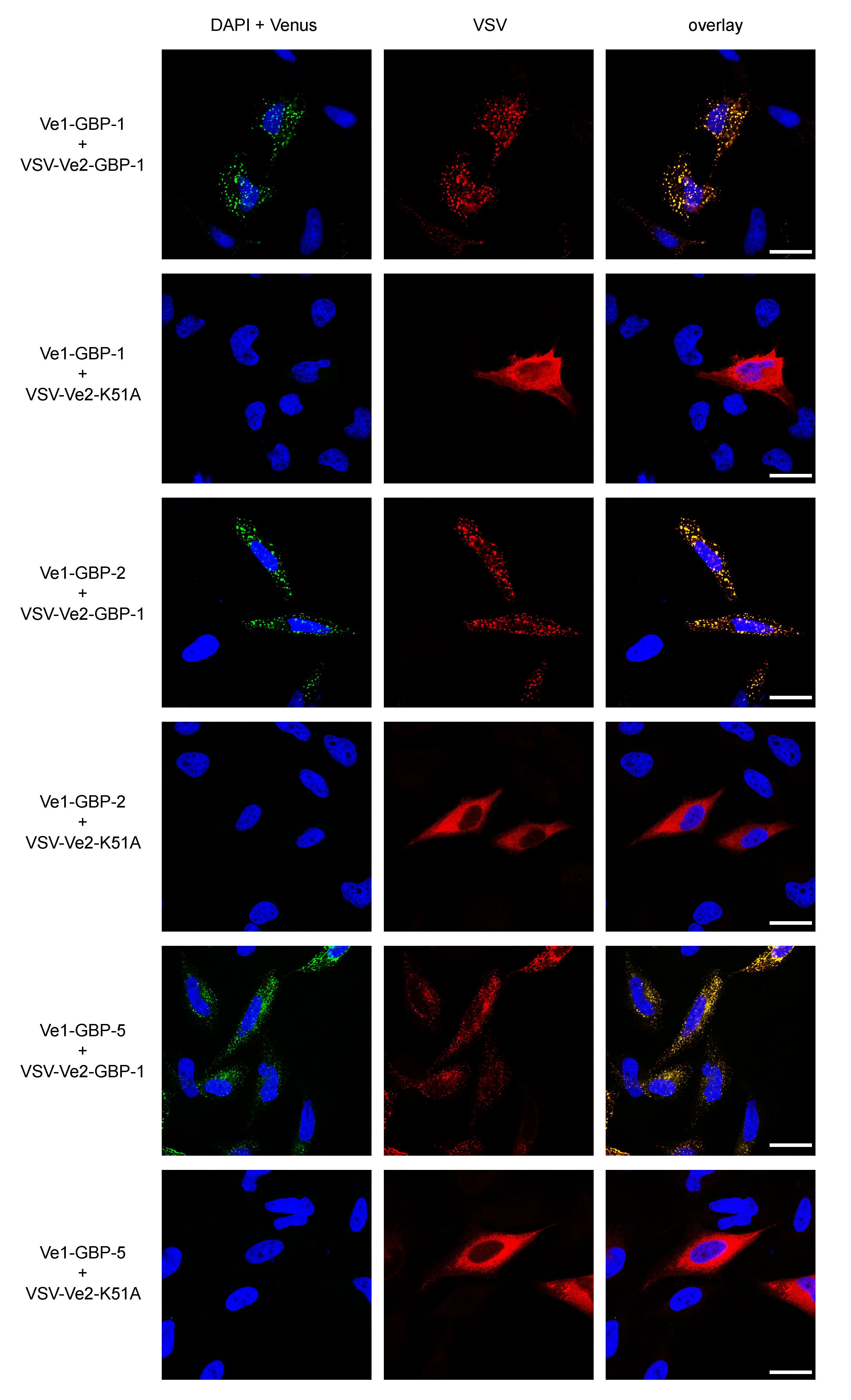

Supplement: Figure S6 — Reciprocal fluorescence complementation assay. HeLa cells were co-transfected with plasmids expressing Venus 1-GBP-1, -GBP-2 or -GBP-5 and VSV-Venus2-GBP-1 K51A. VSV-Venus2 fusion proteins were stained with an anti-VSV antibody and an anti-mouse-AlexaFluor 546 secondary antibody, and nuclei were counterstained with DAPI. Scale bars = 25 µm. (8.71 MB TIF) [file pone.0014246.s007.tif]

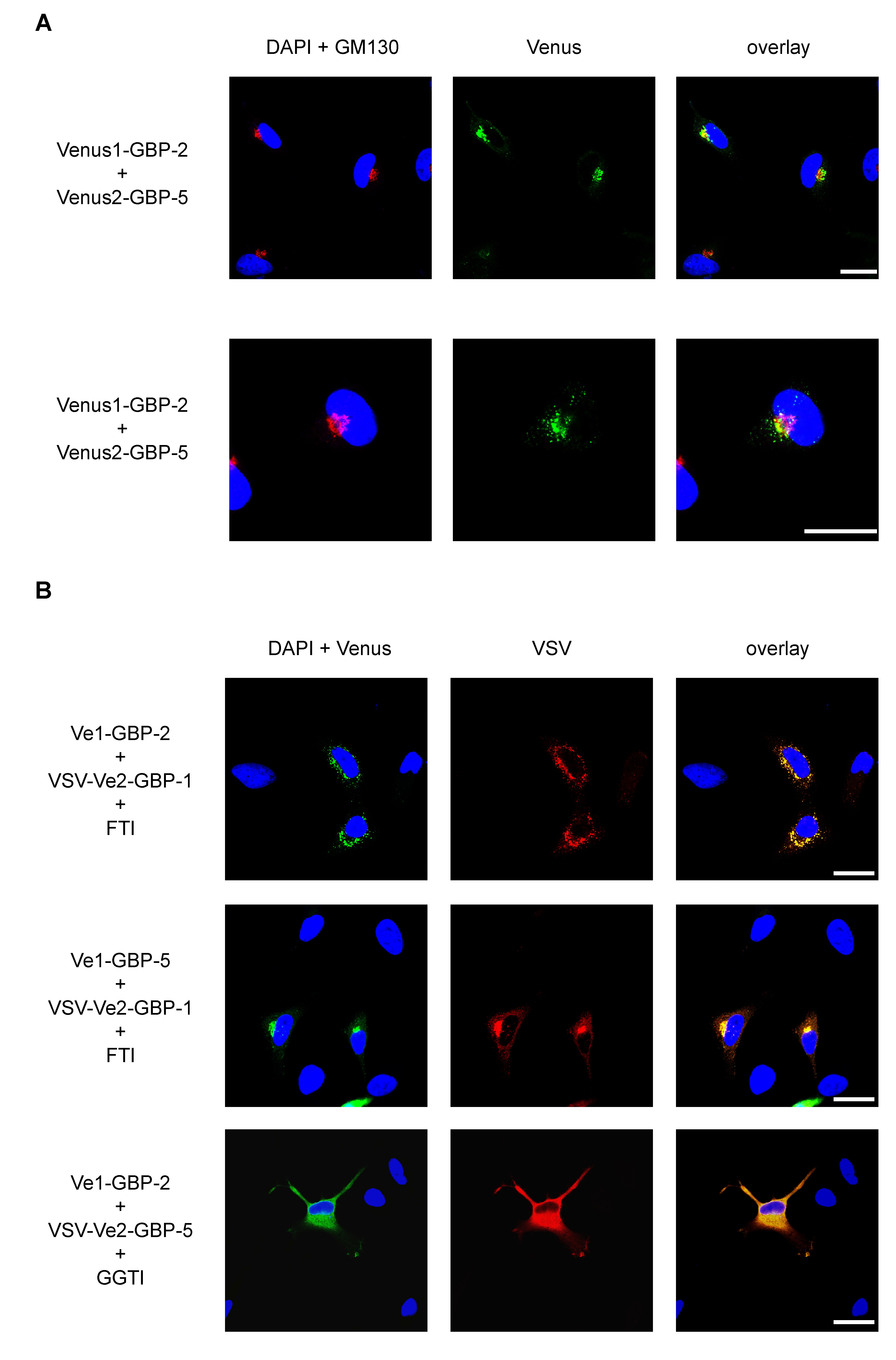

Supplement: Figure S7 — Prenylation-dependent localization of GBP heterodimers.(A) Heterodimers of GBP-2 and GBP-5 localize at the Golgi. HeLa cells were co-transfected with plasmids expressing Venus1-GBP-2 and VSV-Venus2-GBP-5. Cells were stained with an anti-GM130 antibody and an anti-mouse-AlexaFluor 546 secondary antibody, and nuclei were counterstained with DAPI. (B) Prenylation is necessary for membrane association of heterodimers of GBPs. HeLa cells were co-transfected with the expression plasmids of Venus1-GBP-2 or -5 and VSV-Venus2-GBP-1. The reciprocal experiments were performed with Venus-1-GBP-2 and Venus1-GBP-5. VSV-Venus2 fusion proteins were stained with an anti-VSV antibody and an anti-mouse-AlexaFluor 546 secondary antibody, and nuclei were counterstained with DAPI. Cells were treated with 10 µM GGTI or 10 µM FTI when indicated. Scale bars = 25 µm. (5.06 MB TIF) [file pone.0014246.s008.tif]

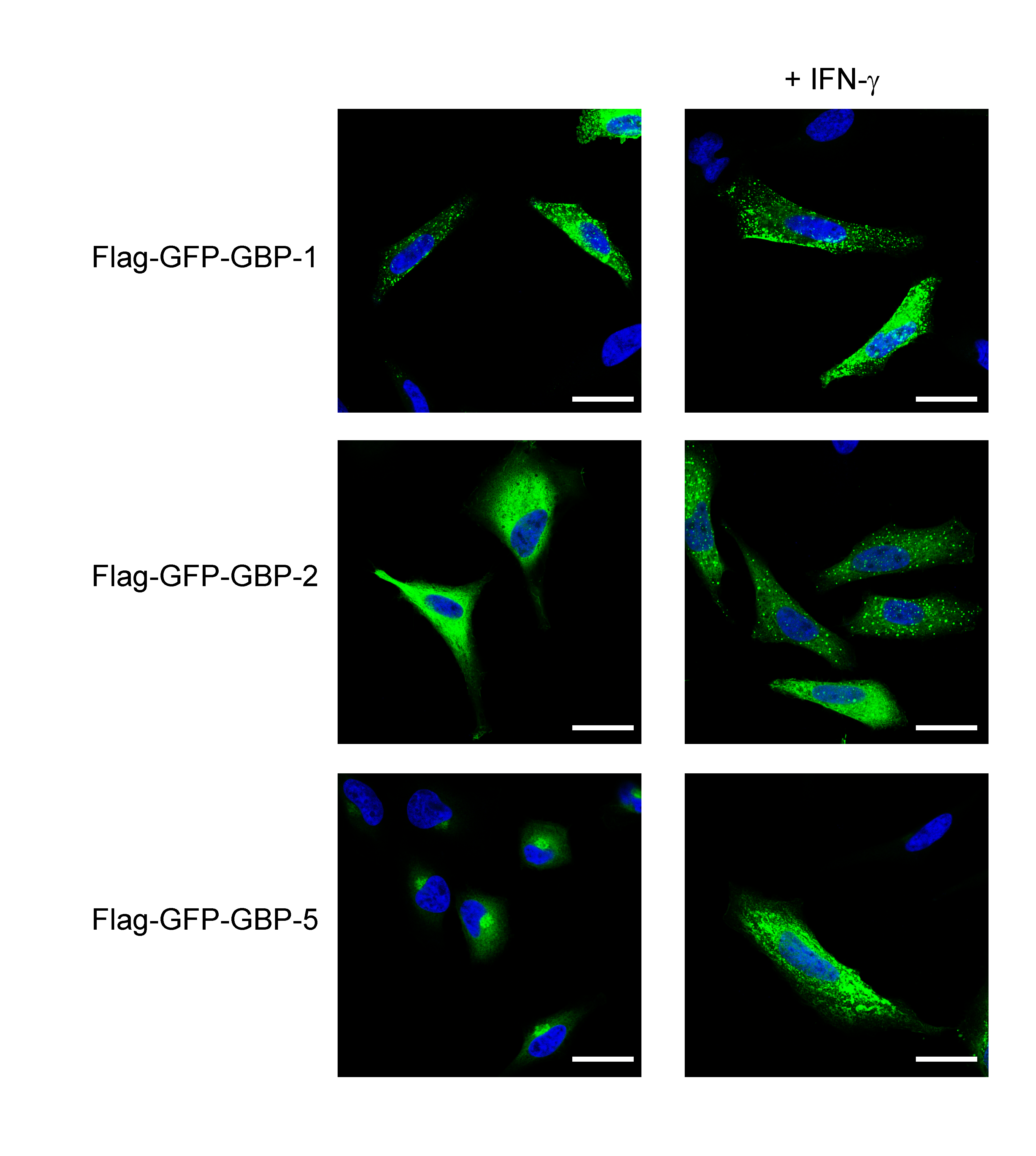

Supplement: Figure S8 — Redistribution of ectopically expressed GBPs in presence of IFN-γ. HeLa cells were transiently transfected with Flag-GFP-GBP-1, Flag-GFP-GBP-2 and Flag-GFP-GBP-5. Cells were treated with 100 U/ml of IFN-γ as indicated. Nuclei were counterstained with DAPI. Scale bars = 25 µm. (3.95 MB TIF) [file pone.0014246.s009.tif]

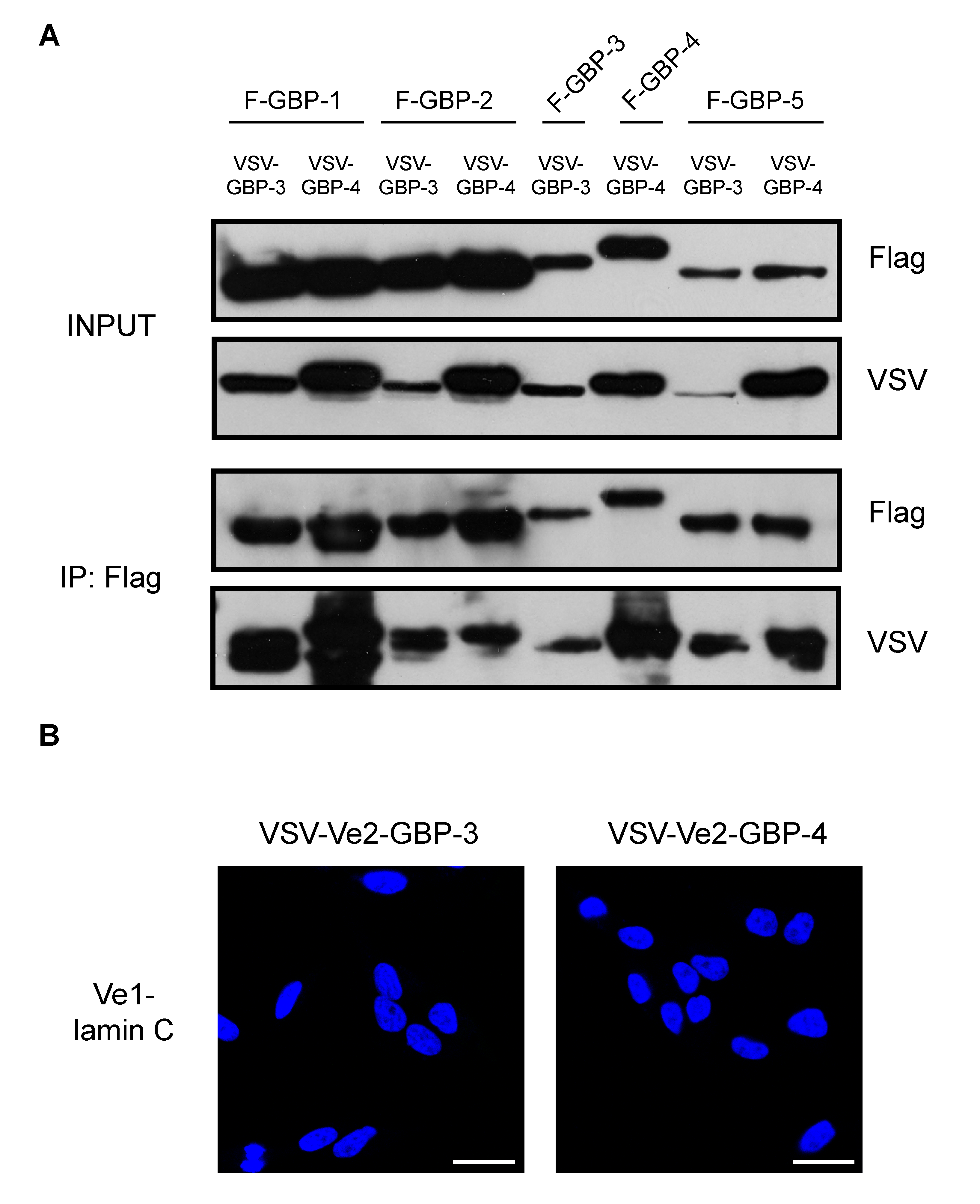

Supplement: Figure S9 — Homo- and heterodimerization of GBP-3 and GBP-4. (A) GBP-3 and GBP-4 are able to homodimerize and to heterodimerize. HeLa cells were co-transfected with Flag-GBPs together with VSV-GBPs, as indicated. Protein extracts were immunoprecipitated with an anti-Flag affinity gel and subjected to western blot analysis. For each co-transfection, cell lysates (10 µg, INPUT) and IP eluates (1∶4 for Flag-detection and 3∶4 for VSV detection) were analyzed. (B) GBP-3 and GBP-4 do not dimerize with lamin C. HeLa cells were co-transfected with plasmids expressing Venus1-lamin and VSV-Venus2-GBP-3 or -GBP-4. Nuclei were counterstained with DAPI. Scale bars = 25 µm. (3.66 MB TIF) [file pone.0014246.s010.tif]
